# The association between social capital and quality of life among a sample of Iranian pregnant women

**DOI:** 10.1186/s12889-019-7848-0

**Published:** 2019-11-09

**Authors:** Masoumeh RezaeiNiaraki, Sadaf Roosta, Zainab Alimoradi, Kelly-Ann Allen, Amir H. Pakpour

**Affiliations:** 1 Students’ research committee, School of Nursing & Midwifery, Qazvin University of Medical Sciences, Qazvin, Iran; 2 Social Determinants of Health Research Center, Research Institute for prevention of Non-Communicable Diseases, Qazvin University of Medical Sciences, Bahonar blv., Qazvin, 34197-59811 Iran; 3 Educational Psychology and Inclusive Education, Faculty of Education, Monash University and The Centre for Positive Psychology, The Melbourne Graduate School of Education, The University of Melbourne, Parkville, Australia; 4 Department of Nursing, School of Health and Welfare, Jönköping University, Jönköping, Sweden

**Keywords:** Pregnancy, Social capital, Quality of life, Mental health, Physical health

## Abstract

**Background:**

Quality of life (QoL) is a multidimensional concept that is affected by various factors. According to the literature, social capital is one of the key determinants of QoL that improves the living conditions of the entire community. This study aimed to investigate the association between social capital and QoL in pregnant women.

**Methods:**

This cross-sectional study included 240 pregnant women with a mean age of 27.98 years who were referred to healthcare centers in Qazvin, Iran. A two-stage random sampling method was used to select the health centers and participants. Social capital, QoL, demographic and obstetric characteristics were assessed.

**Results:**

The mean scores of social capital, physical and mental dimensions of quality of life were 67.43, 70.2 and 71.88 respectively. All dimensions of social capital except for family and friends’ connection and tolerance of diversity had positive significant correlations with the physical and mental health dimensions of quality of life (*r* = 0.17 to 0.28 *p* < 0.05). A univariate regression model revealed that social capital had a significant association with both the physical health (B = 0.40, 95% CI: 0.19–0.61, *p* < 0.001) and mental health (B = 0 .44, 95% CI: 0.18–0.58, p < 0.001) dimensions of pregnant women’s quality of life. In the adjusted model, each unit increase of social capital increased pregnant women’s QoL in both the physical health and mental health dimensions.

**Conclusion:**

Social capital has a significant association with women’s QoL during pregnancy. Therefore, QoL during pregnancy could be improved by considering physical, psychological and social components of their healthcare.

## Introduction

Pregnancy creates unique physiological changes in the body that influence metabolic, hormonal, cardiovascular, respiratory, and musculoskeletal fluctuations more than any other physiological event [1]. The anatomical, physiological, and biochemical changes that occur during pregnancy can be profound, yet entirely normal [2]. Such physical and psychological changes during a healthy pregnancy can affect physical performance, mental health and perceptions of quality of life (QoL) [3–5].

QoL is recognized as a useful construct in health and social care research [6]. As a subjective and multidimensional concept, QoL can be valued and perceived differently by people based on their age, gender, health status, and cultural factors. Research has categorized QoL into five domains, including health, security, appropriate social associations, the right to have a good life, and the right to choose, suggesting that it relates to social and natural capital [7].

Since QoL is a multidimensional concept, various factors can impact people’s perceptions of it, including life stages, lifestyles, personalities, attitudes, social capital, social protection, and socioeconomic conditions. Therefore, QoL cannot be assessed without considering social-level attributes and factors [8].

Social capital is a relatively new concept that’s been used in an increasing number of fields, due to its impact on the living conditions of entire communities [9]. While the QoL of pregnant women has been studied in previous literature, there have only been a few studies that have examined the impact of social capital and health on QoL during pregnancy. Lamarca et al. (2013) reported that women’s high self-rated health, both during the pregnancy and 6 months afterwards, was positively associated with higher levels of individual social capital (i.e., social support and social networking). However, interestingly, neighbourhood social capital (e.g., social control, social trust, neighbourhood security and political trust) had no effect on women’s health [10]. Similarly, Tofani et al. (2015) found that low individual social capital predicted health-compromising behaviors (e.g. smoking, drinking alcohol, and malnutrition) in a sample of pregnant Brazilian women in antenatal care units, while low neighbourhood social capital only predicted inadequate diet [11]. Both studies demonstrate the importance of individual social capital for pregnant women signalling the importance of individual factors in their health care [11, 12]. Contrarily however, Agampodi et al. (2017) found that the domestic and neighbourhood dimensions of social capital were more commonly expressed by pregnant women in Sri Lanka and structural social capital was observed within the micro-communities of the female participants. In the study social support was reported to be low and reserved to family and close friends [12]. Therefore, the few studies that have evaluated the role of social capital during pregnancy indicate mixed findings and these discrepancies warrant further investigation with contextual and cultural considerations in mind.

Social capital plays an important role in people’s ability to access healthcare services, and as consequence, their health [13]. In pregnancy, higher social capital may increase access to maternity care and facilitate healthy pregnancy [14, 15]. Although the goals of prenatal care may vary according to context and service provider, a woman’s individual factors should also be considered [16]. Given that individual social capital is a key determinant of QoL, and that both constructs have been found to be associated with positive health outcomes [6], the aim of this study was to investigate the association between social capital and QoL in pregnant women and contribute to the gap in literature on these constructs.

## Methods

### Study design and setting

This cross-sectional study was conducted between May and December 2018. The participants were pregnant women referred to healthcare centers for prenatal care in Qazvin, Iran. All participants were married. A total of 37 urban health centers in Qazvin were used in the present study. In 2018 approximately 82% of health centers provided prenatal care in Qazvin.

Inclusion criteria for study participants included having a singleton intrauterine pregnancy with a gestational age higher than 10 weeks, and willingness to participate in the study. A history of chronic medical problems (e.g., diabetes, cardiovascular disease, and chronic kidney disease) or psychological problems (e.g., traumatic and stressful incidents within the last 3 months) led to the exclusion of the participants from the study. Having singleton pregnancy and gestational age was confirmed based on participants’ sonogram. As part of routine prenatal care, all of women who attend for prenatal care are sent for sonogram assessment to confirm pregnancy and gestational age. Gestational ages less than 10 weeks were excluded because they are at higher risk of abortion. Multiple pregnancies and having chronic conditions were excluded because experiencing such situations might attract special attention. So these conditions were excluded to have opportunity to investigate what most of women experience in normal pregnancy.

### Sampling

Two stages of random sampling methods were used to select healthcare centers and participants. The first stage involved random cluster sampling. For this purpose, researchers divided Qazvin into five geographical regions: north, south, east, west, and central. Then they randomly chose two health centers from each region. Random sampling was performed using a list of potential participants from the selected centers. A total of ten centers were selected, and 24 pregnant women were randomly recruited to participate in the study.

The sample size was calculated by using the correlation coefficients reported in previous studies. In the study of Rajabi et al. (2013), there was a significant association between general social capital and overall QoL (r = 0.15) [17]. The required number of participants was estimated to be 180 people (r = 0.15, α = 0.05, β = 0.20). With a 30% probability of participants not completing the questionnaires, a total of 240 participants were recruited for this study.

### Definition of variables and measurement

In this research, the variables of social capital, QoL, demographic, and obstetrics were assessed using the following measures:

### Social capital

The Onyx- Bullen Social Capital Questionnaire is a 36 items measure used in the study [18]. It had a Likert scale from “never” to “always”—with scores ranging from 1 to 4. The total score is the sum of all items, and higher scores represent higher social capital. This questionnaire consisted of eight domains: community connections (7 items), social agencies (5 items), trust and safety (5 items), neighbourhood connections (5 items), family & friends (3 items), tolerance of diversity (3 items), value of life (3 items), and work connections (5 items). The face, content, and structure (i.e., through exploratory factor analysis and exploratory causal path analysis) of its validity was confirmed and reported a Cronbach’s alpha coefficient of .84 [18]. The psychometric properties of the Farsi version of this questionnaire was first provide by Eftekharin et al. (2016). In the present study, the cluster correlation coefficient for the subscales scales were more than 0.70, and the Cronbach’s alpha coefficient for the whole scale was 0.96 [19]. It should be noted that the last five items of this questionnaire were specifically associated with work connections. Since the majority of participants identified as “housewives” (i.e., without formal employment/involved in home duties), it was impossible to examine this subscale for all participants. Therefore, the final analysis of this section was performed using 31 items within seven subscales. The Cronbach’s alpha coefficient of 0.82 showed the reliability of this in this study.

### Quality of life

QoL was measured by using the Short Form (36) Health Survey (SF-36). The SF-36 is a well-known health-related instrument for QoL that was developed in the USA [20]. The SF-36 is a general QoL instrument that measures eight health-related concepts, including physical functioning (PF-10 items), Physical role functioning (RP-4 items), body pain (BP-2 items), general health perceptions (GH-5 items), vitality (VT-4 items), social functioning (SF-2 items), emotional role functioning (RE-3 items), and perceived mental health (MH-5 items).

Raw scores were transformed to a 0–100 scale—with higher scores indicating better QoL [20]. The SF-36 has two main dimensions: physical health (PCS) and mental health (MCS) [21]. This tool was translated and validated by Montazeri et al. (2005) in Iran [22]. The Cronbach’s alpha coefficient of 0.87 showed the reliability of this in this study.

### Demographic & Obstetrics Questionnaire

This questionnaire included items about the age, educational level, and occupation of the pregnant woman and husband. It also asked each participant about household socioeconomic status, place of residence, home ownership, duration of residence in the last place of residence, and pregnancy specifics (e.g., the current gestational age, the gender of the fetus, and the number of pregnancies, deliveries, abortions, unwanted pregnancies, and children). The questions were prepared based on the objectives of the study and review of the literature. For content validity, five nursing and midwifery faculty members reviewed and approved it.

Sample materials used for data gathering purpose in this study is provided as Additional file 1.

### Statistical analysis

The SPSS software version 24 and MLwiN Version 2.27 were used to analyze the data. Quantitative variables were reported based on their mean and standard deviation, and qualitative variables were described by their frequency and percentages. The association between the different dimensions of social capital (with physical and mental health dimensions of QoL) was investigated using the Pearson correlation coefficient.

Further assessment of the association between social capital and QoL was examined using the multilevel statistical modelling [23] via a two-level linear regression model, with pregnant women (*n* = 240) at level 1 and centres (*n* = 10) at level 2 using the maximum likelihood method (IGLS). In the regression model, physical component (PCS) and mental component (MCS) of QoL were entered as dependent variables and the total score of social capital was entered as independent variable. Before running the regression model, normality of dependent variable (social capital total score) was assessed using two methods of histogram and Kolmogorov Smirnov test. Kolmogorov Smirnov test with *p*-value of 0.06 verified the normality of this score. Also histogram confirmed this assumption. The regression model was adjusted for potential confounders of demographic characteristics (age, education, socioeconomic status, income and duration of residence in the current place of living) and obstetrics variables (gestational age, number of pregnancies and delivery type, abortion, fetus gender, desire to be pregnant). In the regression model, independent variables with more than two categories were defined as dummy variables. The ICC was calculated for each model. The significant level was considered as < 0.05. The datasets used and analyzed during the current study is provided as Additional file 2.

### Ethical considerations

The current research was approved by the research council of the Faculty of Nursing and Midwifery of Qazvin University of Medical Sciences, Iran. The Ethics Committee of Qazvin University of Medical Sciences reviewed the proposal and approved it under the code IR.QUMS.REC.1397.043. Written informed consent was obtained from all participants after the goals of the study were explained and handling of collected data to ensure privacy and confidentiality was understood.

### Research procedure

After obtaining necessary permissions, the researchers referred to the selected health centers. After introducing the aim and objectives of the study, pregnant women were selected based on the inclusion criteria. After acquiring written informed consent, the participants were asked to fill out the study questionnaires during one of the pregnancy visits. Data was collected at one stage using the interview method. All interviews were done by two of the researchers.

### Findings

#### Demographic characteristics

The researchers invited 240 pregnant women to participate in this study. All of the participants voluntarily filled out questionnaires. The findings showed that the mean (standard deviation) of the women’s age was 27.98 (5.18) years and of their spouses was 32.72 (5.83) years. The majority of the participants had a college diploma (40%) but currently identified as housewives (87.5%). A total of 38.4% of spouses had diplomas, and only 2.9% were completely unemployed. For 79.6% of participants, the family income was between 10 and 30 million Rials, and half of the participants described their socioeconomic situation as weak. More than half of the women (59.6%) had stayed in their current residence for less than 2 years.

In terms of obstetrics characteristics, most participants (44.2%) were experiencing their first pregnancy, half of them had a history of previous childbirth and 80% had no history of abortion. The mean (standard deviation) of the women’s gestational age was 23.5 (8.13) weeks. The majority of them (75.8%) reported wanting the pregnancy and were in their second trimester (55.4%). More details are provided in Table 1.

**Table 1:** Distribution of social capital score according demographic and obstetrics variable

Demographic Variables	No (%)
Women’s educational status	
Under diploma	57 (23.8)
Diploma	96 (40)
Academic	87 (36.2)
Spouse’s educational status	
Under diploma	62 (25.8)
Diploma	92 (38.4)
Academic	86 (35.8)
Women’s Job	
Housewife	210 (87.5)
Employed	30 (12.5)
Spouse’s Job	
Unemployed	7 (2.9)
Employed	233 (97.1)
Household’s income	
< 10 million Rials	45 (18.8)
10–30 million Rials	191 (79.6)
> 30 million Rials	4 (1.6)
Perceived Socioeconomic Status	
Week	122 (50.8)
Moderate	79 (32.9)
Good	39 (16.3)
Period of residency in the current home	
< 2 years	143 (59.6)
2–5 years	68 (28.3)
> 5 years	29 (12.1)
Gravid (No of pregnancy)	
1–2	182 (75.9)
3–4	56 (23.4)
> 4	2 (0.8)
Parity (No of deliveries)	
Nulliparous	122 (50.8)
Multiparous	118 (49.2)
No of Abortion	
0	192 (80)
1	37 (15.4)
2	11 (4.6)
No of lived child	
0	122 (50.8)
1	87 (36.3)
2	28 (11.7)
3	3 (1.3)
Pregnancy willingness	
Wanted	182 (75.8)
Unwanted	58 (24.2)
Fetus gender	
Female	84 (35)
Male	90 (37.5)
Not know	66 (27.5)

#### The association between social capital and QoL among pregnant women

The mean (standard deviation) of social capital was 67.43 (12.48). The total score of social capital was normally distributed among the sample. The mean score for QoL in the physical health dimension was 70.2 (21.06) and in the mental health dimension 71.88 (20.66) (Table 1). The association between different dimensions of social capital with physical and mental health dimensions of QoL is shown in Table 2. All dimensions of social capital except family and friends’ connection and tolerance of diversity had positive significant associations with the physical and mental health dimensions of the QoL.

**Table 2:** correlation between Social capita subscales and total scores with physical health (PCS) and mental health (MCS) dimensions of QOL

		Community Connection	Social Agency	Trust Safety	Neighborhood Connection	Family & Friends connections	Tolerance of Diversity	Value of Life	Social capita total score
PCS	r	.24	.18	.17	.19	−.08	.10	.21	.24
	*p*-value	>.001	.004	.007	.003	.221	.125	.001	> 0.001
MCS	r	.28	.19	.22	.21	−.05	.08	.21	.26
	*p*-value	>.001	.004	.001	.001	.488	.242	.001	> 0.001

Findings of the association between social capital and QoL using the linear regression model are provided in Table 3. The regression model showed that social capital had a significant association with both the physical and mental health dimensions of the QoL among pregnant women. The association of social capital on pregnant women’s QoL was significant after adjusting for demographic and obstetrics characteristics. In the adjusted model, each unit increase of social capital in pregnant women resulted in an increase in physical health (0.40) and mental health (0.44) (Table 3).

**Table 3:** Results of regression analysis regarding the association of social capita with physical health (PCS) and mental health (MCS) dimensions of pregnant women’s quality of life

Model	Unstandardized Coefficients			Sig.	95.0% Confidence Interval for B		
	B	Std. Error			Lower Bound	Upper Bound	
Social Capita	MCS-QOL						
Unadjusted Model	.438	.103		<.001	0.235	0.641	
Adjusted Model^a^	.407	.099		<.001	0.212	0.602	
Intercept	42.355	7.070		<.001	28.426	56.284	
${\hat{\upsigma}}_{\mathrm{st}}^2$ (pregnant women)	395.434	36.098		<.001	324.314	466.554	
${\hat{\sigma}}_{sc}^2$ (centres)	22.143	16.381		0.174	−10.131	54.417	
	PCS-QOL						
Unadjusted Model	.399	.106		<.001	0.190	0.608	
Adjusted Model^a^	.366	.100		<.001	0.169	0.563	
Intercept	66.038	12.578		<.001	41.257	90.819	
${\hat{\upsigma}}_{\mathrm{st}}^2$ (pregnant women)	353.841	32.301		<.001	290.200	417.479	
${\hat{\sigma}}_{sc}^2$ (centres)	12.638	9.247		0.172	−5.580	30.856	

^a^Adjusted for demographic and obstetric characteristics

## Discussion

QoL is a multidimensional concept that is influenced by various factors, including social capital [8]. In the conceptual model of the World Health Organization for the social determinants of health, social capital is considered to be a determinant of health and well-being for individuals and communities [24]. The results of the present study showed that the mean score of the participants’ QoL was 70.2 (21.06) for the physical health dimension, and 71.88 (20.66) for the mental health dimension. In previous Iranian studies, researchers reported similar findings about the QoL of pregnant women. For instance, Moafi et.al (2018) reported that the mean score for QoL in pregnant women was between 64 (23.4) for vitality and 76.4 (21.1) for social functioning [25]. Similar findings were also reported by Abbaszadeh [26] and Azizi [27].

The main purpose of this study was to examine the association between social capital and QoL in pregnant women. The results suggested a positive and significant correlation between social capital and the physical (0.24) and mental health dimensions (0.26) of QoL. Although the association between QoL and social capital in pregnant women had not previously been studied, positive and significant associations have been reported between social capital and QoL (as well as other aspects of health in other age and social groups) [6, 8, 28–31]. For example, in a study by Rajabi et al. (2013), the Pearson correlation coefficient between the total social capital of teachers (both male and female)—including the physical and mental components of QoL—was 0.14 [17]. Therefore, the findings of the present study showed that while women undergo substantial physical and psychological changes during pregnancy, their social capital can have a positive effect on their QoL. In recent years, the protective effect of social capital on mental health has been increasingly respected by researchers. In 2013, Bouchard reported that a lack of social capital is associated with mental health problems [32]. In a cross-sectional study, De Silva et al. (2007) found an inverse association between cognitive social capital with mental illness in four low-income countries [33]. Similarly, Akbari et al. (2017) found that social capital and perceived social support explained 30% of the variance of mental health in women [29].

For pregnant women, social capital has had a positive effect on mental health. George et al. (2013) reported that higher social capital level was associated with lower scores in Edinburgh’s postpartum depression scale among pregnant women—from 24 weeks of gestation to 10 weeks postpartum [34]. Zhou et al. (2017) reported a significant association between pregnancy-related depression and social trust (ST), social reciprocity (SR), social networking (SN), and social participation (SP) [35].

Social capital can affect the psychological mechanisms of the body, and it can consequently affect an individual’s mental health [36]. Having healthy social relationships and feeling socially supported can be a protective factor against stress and psychological distress [37]. In addition to the impact of social capital on psychological health, it can be a significant determinant of physical health too [38]. Social capital also has a potential role in modifying health-related behaviours, including diet, sleep, physical activity, alcohol consumption, smoking, and other substance abuse [38]. Researches have proposed various mechanisms for the influence of the components of social capital on health outcomes. Health-related behaviours and lifestyle are often social and cultural phenomenon derived from the combination of individual behaviours and living conditions as primary mediators [38]. Health-related behaviours are developed in the interaction of the social environment with individuals’ psychological and biological characteristics [39].

For the present study, a positive and significant association was observed between social capital and the physical and mental health dimensions of QoL for pregnant women. It seems that the components of social capital (including community connection, social agency, trust, safety, neighbourhood connection, and value of life) can improve the physical and mental health dimensions of QoL during pregnancy. According to the regression model in the present study, after controlling for demographic and obstetric characteristics, an increase in social capital during pregnancy can increase the physical health dimension of QoL by 0.40, and the mental health dimension by 0.44.

Notably, in the present study, connections with family and friends and the tolerance of diversity did not have significant correlations with the physical and mental health dimensions of QoL. One explanation for this may be that people tend to live further away from family relatives then they did in decades past. Therefore, long-distance relationships can diminish the role(s) of family members in social networks. If people do not rely on their families for support, especially if distance is a barrier, these connections may have a smaller impact on their social capital.

The lack of a significant association between the tolerance of diversity and the QoL of pregnant women might be able to have multiple explanations that warrant further research. Probable reasons include the impact of cultural contexts and the preference to live and socialise with culturally similar people. As Onyx and Bullen (2000) and Hyyppä (2010) stated, the association between social capital and health-related behaviors depends largely on cultural, social, and historical context [18, 38].

## Limitations

This research examined the association between the components of social capital and QoL in pregnant women providing early empirical support that warrants future research. One of the most important limitations of the present study is its cross-sectional design. While this kind of study can provide valuable information about the overall status of study variables, it cannot provide information about the direction of the associated variables. Therefore, longitudinal studies can draw a more precise picture of the role of social capital on various aspects of individual health during pregnancy. Also, researchers used a self-report method for this data collection.

Therefore, collecting objective health information (e.g., maternal weight indices, the number of referrals for care during pregnancy, the onset of prenatal care, the birth weight, the gestational age at birth, and other consequences of pregnancy and childbirth) can provide more data on the way social capital, pregnancy, and childbirth are associated.

## Conclusion

Social capital has a significant positive association on QoL during pregnancy. Clinicians and practitioners working in prenatal care should be mindful of the importance of social capital in improving the QoL of women during pregnancy.

## Supplementary information

**Additional file 1.** Sample Questionnaires; Sample materials used for data gathering purpose in this study is provided.

**Additional file 2.** Raw dataset. The datasets used and analyzed during the current study is provided.

## Data Availability

Data and materials are provided as Additional files 1 and 2.

## Abbreviation

QoL: Quality of life
