# Supplementary material for: The association between social capital and quality of life among a sample of Iranian pregnant women
Source: BMC Public Health. 2019 Nov 9;19:1497. doi: 10.1186/s12889-019-7848-0 (PMC6842465; doi:10.1186/s12889-019-7848-0)
Supplement: Supplementary file 1 — Additional file 1. Sample Questionnaires; Sample materials used for data gathering purpose in this study is provided. [file 12889_2019_7848_MOESM1_ESM.docx]

Dear Mother,

The following questions relate to the research project, " ***The association between social capital and quality of life among a sample of Iranian pregnant women***". The project was approved by the ethical committee of Qazvin University of Medical Science, Qazvin, Iran. This project aimed to investigate the impact of perceived social capital on pregnant women's quality of life. Identifying the relationship between quality of life and social capital during pregnancy, may guide care and support strategies for clinicians and health care professionals.

You are invited to participate in this project. All information gathered will remain anonymous, therefore your privacy and confidentiality will be protected. You may freely respond to answers and cease participation in the study at any time. If you decline participation in this research there will be no change to your existing care routine.

| **Part 1- Demographic characteristics.** | | | | |
| --- | --- | --- | --- | --- |
| How old are you? | --------- year |  | |  |
| How old is your husband? | --------- year |  | |  |
| What is your educational status? | Under Diploma🗆 | Diploma🗆 | | Academic 🗆 |
| What is your spouse's educational status? | Under Diploma🗆 | Diploma🗆 | | Academic 🗆 |
| What is your job? | Employed 🗆 | Housewife 🗆 | |  |
| What is your spouse's job? | Un employed🗆 | Employed 🗆 | |  |
| How much is your household income? | < 10 million Rials🗆 | 10-30 million Rials🗆 | | > 30 million Rials🗆 |
| Where do you live? | Rural | Urban | |  |
| Do you own home? | No | Yes | |  |
| How do you rate your family’s socio-economic status? | Good | Moderate | | Weak |
| Period of residency in the current home | ----------------months | | | |
| **Part 2- Obstetrics** **characteristics** | | | | |
| Gestational age based on last menstrual period or 1st trimester sonogram: -------------- weeks | | | | |
| Obstetrics history | How many times you experience Pregnancy? ------ How many times you experience childbirth? -----  How many times you experience Abortion? ----- | | | |
| How many children do you have? |  | | | |
| Do you want this pregnancy? | No 🗆 | Yes 🗆 | | |
| Fetus gender | Don't Know 🗆 | Female 🗆 | Male 🗆 | |
| **Part 3- Short Form (36) Health Survey (SF-36)** | | | | |
| **Part 4- Social capital Questionnaire developed by Onyx- Bullen** | | | | |
